# Supplementary material for: Fungal Host Affects Photosynthesis in a Lichen Holobiont
Source: J Fungi (Basel). 2022 Nov 30;8(12):1267. doi: 10.3390/jof8121267 (PMC9784818; doi:10.3390/jof8121267)
Supplement: Supplementary file 1 [file jof-08-01267-s001.zip › jof-1951626-supplementary.pdf]

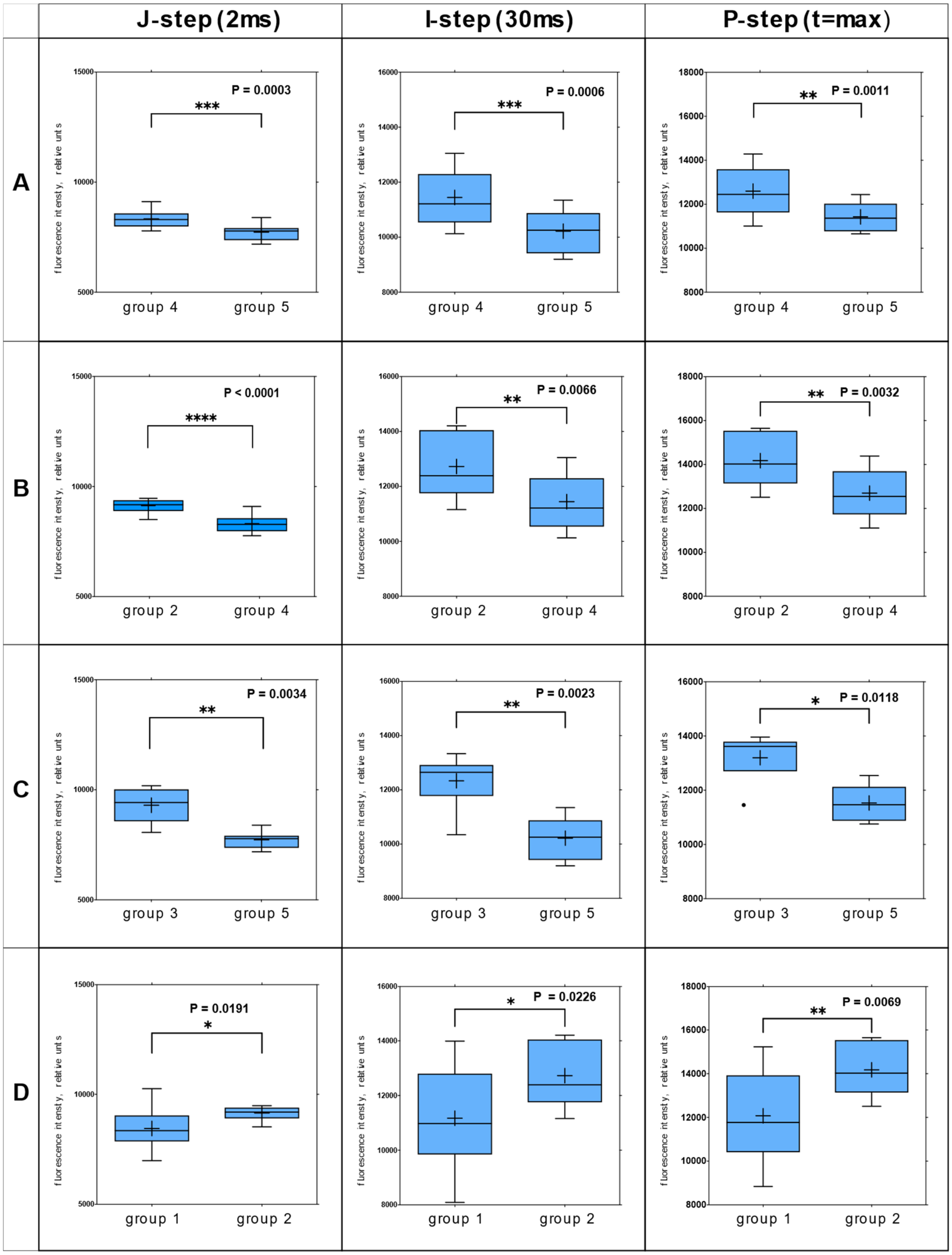

Figure S1. Fluorescence intensities for the J-, I- and P-step for the comparisons of the chlorophyll a fluorescence rise kinetics (O-J-I-P-transients) for Figure 1. Significance for each comparison of the curves for each is shown as p-value ($p<0.05$). Analysis based on a t-test with Welsh-correction.

Table S1. Brown-Forsythe and Welsh ANOVA adjusted p-Value correlation for the J-step (A) as well as the I-step (B) for the O-J-I-P transients of figure 1.

**A**	Group 1	Group 2	Group 3	Group 4	Group 5
Group 1		0,4523	0,7951	>0.9999	0,4536
Group 2	0,4523		>0.9999	<0.0001	<0.0001
Group 3	0,7951	>0.9999		0,4142	0,0761
Group 4	>0.9999	<0.0001	0,4142		0,0145
Group 5	0,4536	<0.0001	0,0761	0,0145	

**B**	Group 1	Group 2	Group 3	Group 4	Group 5
Group 1		0,555	0,9516	>0.9999	0,9652
Group 2	0,555		>0.9999	0,2257	0,0008
Group 3	0,9516	>0.9999		0,8795	0,0629
Group 4	>0.9999	0,2257	0,8795		0,0296
Group 5	0,9652	0,0008	0,0629	0,0296	

Material and methods supplementary

Sampling site and sampling design

Photosynthetic performance analysis

All thalli for the photosynthetic performance analysis were collected from the site at ~1500 m a.s.l. (population #5 in Fig. S1) because it harbours the highest mycobiont and photobiont diversity [see Fig. 5 in 28]. Thalli of the two lichen species were collected using a knife - when possible, from the same rock - and placed directly into paper bags.

Mycobiont and photobiont sequencing

To identify intraspecific fungal lineages, we genotyped each sample (whole lichen thalli) at the fungal single-copy protein coding gene *MCM7* (DNA replication licensing factor mini- chromosome maintenance complex component 7) following Sadowska-Des *et al*. [30]. This locus was chosen because it is the population genetic marker with the highest resolution among commonly used loci in *Umbilicaria* species [28, 30]. For the photobiont, we identified the algal species using the internal transcribed spacer region (ITS) of the rRNA operon. We sequenced the fungal *MCM7* region using the primer pair MCM7FOR2 (f) AGGTGAACGCTTACACATGC; MCM7REV2 (r) CGGGAGCTATGGATCTTGAG [30] and the algal ITS region using the ITS1a/ITS4: FDGITS2-f: AGCGAAATGCGATACGTAGTGT; FDGITS2-r: GGGTGTTCTTGCCTGACCTC primer pair [26].

For both markers, PCR was performed in 25-μl reactions containing 0.65 U TaKaRa ExTaq (Clontech Laboratories Inc., Palo Alto, CA, USA), 2.5 μl of buffer, 18.5 μl of water, 0.5 μl of bovine serum albumin (BSA; 10 mg ml-1), 2.0 μl of dNTP mixture (2.5 mM each), c. 5 ng of total DNA (0.5 μl), and 0.22 lM (0.5 μl) forward and reverse primers. The PCR cycle conditions were as follows: initial denaturation at 95°C for 4 min followed by 35 cycles of 95°C for 30 s, 58°C for 30 s and 72°C for 20 s, and a final elongation at 72°C for 5 min.

Cycle sequencing reactions were as follows: 25 cycles of 20 s 96°C, 5 s 50°C, 2 min 60°C. Forward and reverse strand sequences were detected in an ABI PRISM 3730 DNA Analyzer (Life Technologies). Sequence contigs were trimmed, assembled, aligned and manually edited in CLC DNA Workbench software (CLC-Bio, Mühltal, Germany).

Consensus sequences were compared against the *Umbilicaria* and *Trebouxia* sequence lists from Dal Grande *et al.* [28] using BlastN; positive matches were considered for sequences exhibiting at least 99% of similarity with a full-length *Umbilicaria* or *Trebouxia* sequence.
